# Feasibility of multimodal metabolic analysis for detecting early changes in acute neuroinflammation

**DOI:** 10.1186/s12974-026-03839-7

**Published:** 2026-05-04

**Authors:** James T. Grist, Ilia Evstafev, Dominika Olesova, Signe E. Nynäs, Matej Orešič, Alex M. Dickens, Damian J. Tyler, Yvonne Couch

**Affiliations:** 1 Oxford Centre for Clinical Magnetic Resonance Research, Cardiovascular Medicine, Radcliffe Department of Medicine, University of Oxford, John Radcliffe Hospital, Headley Way, Headington, Oxford, OX3 9DU UK; 2 Department of Radiology, Oxford University Hospitals, Oxford, UK; 3 Department of Physiology, Anatomy, and Genetics, University of Oxford, Oxford, UK; 4 Turku Bioscience Centre, University of Turku and Åbo Akademi, Tykistökatu 6, Turku, FI- 20520 Finland; 5 School of Medical Sciences, Faculty of Medicine and Health, Örebro University, Örebro, Sweden; 6 Department of Chemistry, University of Turku, Tykistökatu 6, Turku, FI-20520 Finland; 7 Nuffield Department of Clinical Neurosciences, Dorothy Hodgkin Crowfoot Building, Sherrington Road, Oxford, OX1 3QU UK

**Keywords:** Imaging, Neuroinflammation, Pyruvate, Hyperpolarized, Metabolism

## Abstract

Given the prevalence of metabolic perturbations in a variety of neurological and neurodegenerative diseases, understanding and monitoring brain metabolism is a key step in our advancement of therapies. The details of the citric acid cycle were established at the beginning of the last century but only recently have its metabolic intermediates been observed *in vivo* in the brain. In this study, we employed orthogonal analyses to investigate metabolic alterations in response to acute neuroinflammation *in vivo*, demonstrating a multi-technique approach that could be used for future studies.

Hyperpolarized [1-^13^C] pyruvate spectroscopy revealed an early decline in pyruvate metabolism via pyruvate dehydrogenase (PDH), leading to reduced ^13^C-bicarbonate formation. This metabolic disruption occurred despite the absence of structural or perfusion changes on conventional MRI. Further analysis of polar metabolites in the ipsilateral hemisphere confirmed ongoing inflammatory processes. These findings highlight the potential of this dual technique approach to inform upon metabolic changes due to neuroinflammation.

Combining methods to probe metabolism in invasive (metabolomics) and non-invasive (hyperpolarized MRI) manners, this represents a promising translational approach for real-time metabolic assessments in an area of the body, the brain, where studying processes such as metabolism has traditionally been challenging. This study has demonstrated the approach to monitor changes in metabolism in response to inflammation in the brain.

**Supplementary Information:**

The online version contains supplementary material available at 10.1186/s12974-026-03839-7.

## Introduction

Current understanding suggests that ongoing inflammation in the brain is an important contributor to the development of neurodegenerative diseases such as dementia, but that it is also a significant factor in neuroinflammatory diseases such as multiple sclerosis (MS) [1–3]. Monitoring on going inflammatory processes [4], and restoring homeostasis, is crucial for minimizing ongoing brain damage after injury, as well as monitoring the efficacy of anti-inflammatory therapies.

The readout of metabolic pathways in the brain may go some way to informing us of ongoing inflammatory pathology, as a close but indirect biomarker of the activated central nervous system (CNS) immune system [5]. For example, astrocytes harness aerobic glycolysis to metabolise glucose to lactate, whereas quiescent microglia and neurons rely upon mitochondrial oxidative phosphorylation (OXPHOS) to fuel adenosine triphosphate (ATP) production through the Tricarboxylic Acid (TCA) Cycle [6]. [1-^13^ C]pyruvate can be metabolised through glycolysis (via lactate dehydrogenase (LDH), with [1-^13^ C]lactate produced) and OXPHOS (via pyruvate dehydrogenase (PDH), hallmarked by ^13^C bicarbonate production). It is possible to monitor these pathways in the brain using hyperpolarized magnetic resonance imaging (MRI) [7, 8]. Hyperpolarized carbon-13 MRI harnesses the transient increase in the available signal from a ^13^C-labelled substrate, often via the process of dynamic nuclear polarization, to allow for the real-time tracking of the metabolic fate of that label *in vivo* [8]. Previous work has demonstrated that macrophage recruitment after cardiac ischemia can be monitored using [1-^13^ C]pyruvate [9], as well as demonstrating increased [1-^13^ C] lactate formation in models of neuroinflammation [10]. One previous study has assessed the metabolic changes associated with lipopolysaccharide (LPS) in the mouse brain, detecting upregulated lactate production at 7 days post injection, correlated with markers of astroglyosis and microglial proliferation [11], demonstrating the potential for the detection of neuroinflammation behind the closed blood-brain barrier.

Clinically, the current technique for monitoring inflammation in the brain is positron-emission tomography (PET). For example, mitochondrial translocator protein (TSPO) has been shown to be upregulated during periods of inflammation in a variety of cells, including astrocytes and microglia [12]. By using radiolabelled ligands, such as ^11^C PK11195, it is possible to image this activity *in vivo* to study inflammation in brain injury [13]. Areas of high TSPO binding have traditionally been thought to represent microglial activation in the brain. However, recent research has demonstrated that whilst this may be true in rodents, it remains unclear whether a similar pattern occurs in humans [14]. Moreover, in some inflammatory diseases, such as MS, there is not a significant upregulation of TSPO and as such, use of this technique to monitor inflammation may be limited [15].

Metabolic imaging, as opposed to imaging specific inflammatory processes, can be carried out using ^18^F(lourine) Deoxy Glucose Positron Emission Tomography (^18^FDG-PET) or Magnetic Resonance Spectroscopy (MRS). FDG-PET uses radiolabelled deoxy glucose which cannot participate in the later stages of metabolism or exit the cell, resulting in images displaying increased signal intensity from metabolically active cells. However, neurodegenerative diseases such as dementia are known to result in metabolically underactive cells [16, 17] meaning that traditional methods such as PET are less sensitive to subtle changes in mitochondrial metabolism, and as such may fail to miss earlier stages of the neurodegenerative process. One of the earlier stages of neurodegeneration may be neuroinflammation [18].

A combined approach harnessing spatial metabolomics, hyperpolarized MR, molecular biology, and histopathology has been demonstrated in the oncological field [19], with results allowing a wide array of pathways to be explored and then a translational, tailored, ^13^C labelled biomarker derived for non-destructive experiments. These orthogonal techniques allow for holistic profiling of the tissue level response to an oncological challenge, and so the primary aim of this project was to translate this combined approach to a model of acute neuroinflammation. This proof-of-concept study will then pave the way for using this approach in more complex neuroinflammatory pathologies.

## Materials and methods

### Animals

All procedures conformed to the Animal (Scientific Procedures) Act (1986) and were approved by the University of Oxford Animal Ethics Committee and the UK Home Office. The studies were conducted, and the manuscript prepared in accordance with the ARRIVE guidelines [20]. All experimental procedures were carried out in the light phase of the animals’ light-dark cycle. Four adult male Wistar-Han rats per group were housed under standard light-dark conditions (12 h) and allowed access to food and water *ad libitum*.

### Surgery

Stereotaxic procedures were carried out as previously described [21]. Animals were anaesthetized using 2% isoflurane in 60:40 oxygen: nitrous and mounted on a stereotaxic frame. The skull was exposed and a burr hole was drilled above the striatum (+ 1 A/P, -2.5 M/L, -4.5D/V from Bregma). 10 ng of lipopolysaccharide (*E.Coli* B26:06; Sigma-Aldrich, UK) in 1 µl of sterile 0.9% saline was injected over a period of 5 min using a glass micro-needle. The needle remained in place for 2 min prior to slow withdrawal. Adsorbable sutures were used to close the wound. Animals were allowed to recover for 24 h prior to imaging and tissue collection.

### Imaging

Animals were anesthetised with 2.5% isoflurane in 60:40% oxygen: nitrous, a tail vein cannula inserted, and reduced to 2% when in the magnet as per a previously established protocol optimised for bicarbonate detection [22]. Animals were placed in a custom-made cradle and placed inside of a 7 T magnet (Agilent magnet, Varian console) with a 2-channel ^13^C coil (Rapid Biomedical, Rimpar, Germany) used for reception and a ^1^H/^13^C volume coil (Rapid Biomedical) for transmit. Approximately 30 µL of [1-^13^ C]pyruvate was prepared with 3 µL of 1:50 gadolinium in water (Dotarem, 1:50 dilution in H_2_O, 3 μ:30 μ diluted solution: [1-^13^ C]pyruvate) and trityl radical (OXO63, 15 mM) and polarized as previously described [22]. After approximately 1 hour of polarization, the sample was dissolved with 4.5 mL sodium hydroxide heated to approximately 175 degrees. Animals were injected with 1 mL of hyperpolarized [1-^13^ C]pyruvate over 10 s, with 200 µL of saline used as a flush, and a slice selective spectroscopy protocol was used to acquire data from each hemisphere (Repetition time per slice = 500 ms, temporal resolution for both slices = 1 s, flip angle = 15 degrees, bandwidth = 5 kHz, slice thickness = 10 mm, slice gap = 2 mm). The ^13^C coil was replaced with a ^1^H 4-channel surface receive coil and T_1_- (pre and post gadolinium infusion, 3D Gradient and RF spoiled gradient echo, Field of View (FOV) = 40 mm^3^, matrix = 256 × 256 × 64, reconstruction matrix = 512 × 512 × 128, Repetition time (TR) = 5 ms, Echo Time (TE) = 1 ms, flip angle = 12 degrees) and perfusion weighted- imaging (200uL Dotarem infused over 0.5 s with 300uL saline flush, 2D Gradient Echo, FOV = 30 mm^2^, slice thickness = 1 mm, acquisition matrix = 128 × 64, reconstruction matrix = 128 × 128, TR = 20ms, TE = 10 ms, Temporal resolution = 1.28 s). Spectra were summed in the time domain and quantified using jMRIUI v5.2, with the fit for each metabolites used to calculate [1-^13^ C]lactate: [1-^13^ C]pyruvate, ^13^C bicarbonate: [1-^13^ C]pyruvate, and ^13^C bicarbonate: [1-^13^ C] lactate. Perfusion data were processed using model free deconvolution, with a region of interest (Supplementary Fig. 1; note the same ROI was used for all imaging and spatial metabolomics) placed at the surgical injection site and on the contralateral side [22]. T_1_ weighted imaging pre- and post- contrast injection were visually assessed for post-contrast enhancement. Images were visually inspected for focal enhancement within the injected hemisphere. No quantitative signal intensity analysis was performed.

### Tissue Processing

All downstream analyses (histology, spatial metabolomics, bulk metabolomics, RNA expression, and enzymatic assays) were performed using tissue obtained from the same imaging cohort of animals. Animals were euthanized and intracardially perfused with cold 0.9% saline containing 10 U/mL heparin. Brains were rapidly removed and snap-frozen within 3 min of euthanasia (mean time to freeze 3 min, range 2–5 min) using isopentane cooled on dry ice to minimize post-mortem metabolic changes. Frozen tissue was stored at − 80 °C until further processing. For histological analysis, 12 μm sections were cut on a cryostat and mounted onto Superfrost™ Plus slides. For molecular analyses, hemispheres were separated into ipsilateral and contralateral regions and processed for RNA extraction and protein/enzyme assays as described below.

### qPCR

RNA was extracted from approximately 25 mg of frozen brain tissue using the Qiagen© RNEasy Mini Kit with QiaShredders. RNA concentration was measured using a NanoDrop (ThermoFisher) and 800 ng of sample was converted to cDNA using the Applied Biosystems High Capacity cDNA conversion kit. Real-time qPCR was performed with duplicates for each sample (15 ng/well) using SYBR green qPCR master mix (PrimerDesign) and the Applied Biosystems QuantStudio Flex 7 with the following cycle conditions: hot start 2 m at 95 °C, 40 cycles of 15 s at 95 °C and 60 s at 60 °C with data acquisition during the 60 °C phase. The following primers were purchased from Merck as Pure&Simple Primers: *IL-1β*: F: CACCTCTCAAGCAGAGCACAG; R: GGTTCCATGGTGAAGTCAAC; *IL-6*: F: TCCTACCCCAACTTCCAATGCTC; R: TTGGATGGTCTTGGTCCTTAGCC. *ICAM*: F: AAACGGGAGATGAATGGTACCTAC; R: TGCACGTCCCTGGTGATACTC; *VCAM*: F: GGCTCGTACACCATCCGC; R: CGGTTTTCGATTCACACTCGT; *PDK1*: F: TACAGAACCAACCACGAGGC R: CCACATTTGGCTTTGCCAGG *PDK2*: F: ACCCAGTCTCCAACCAGAAC; R: GAGATGCGGCTGAGGTAGAA. *PDK3*: F: TCCTAGCGCTCTTGTACCCT; R: CACCCAAGTACCACACCTCC*PDK4*: F: GGATTACTGACCGCCTCTTTAGTT; R: GCATTCCGTGAATTGTCCATC GAPDH: F: GCAAGTTCAACGGCACAG r: CGCCAGTAGACTCCACGAC Relative expression was determined using the Pfaffle method [23] and normalized to the housekeeping gene GAPDH and further normalized to the contralateral hemisphere of the control animals.

### Western blotting

Brain tissue was homogenized in ice-cold lysis buffer (50 mM Tris-HCl, pH 7.4, 150 mM NaCl, 1% Triton X-100, 1% sodium deoxycholate, 0.1% SDS) supplemented with a protease and phosphatase inhibitor cocktail (Roche, Basel, Switzerland). The homogenates were centrifuged at 12,000 × g for 15 min at 4 °C, and the supernatants were collected for protein quantification. Protein concentrations were determined using the bicinchoninic acid (BCA) assay (Thermo Fisher Scientific, Waltham, MA, USA). For each sample, 10 µg of protein was mixed with 4× NuPAGE LDS sample buffer (Invitrogen, Carlsbad, CA, USA) and 10× reducing agent (β-mercaptoethanol; Invitrogen) and heated at 70 °C for 10 min. Proteins were separated on 4–12% Bis-Tris gradient gels (NuPAGE, Invitrogen) using MOPS SDS running buffer (NuPAGE, Invitrogen) under constant voltage (200 V) for approximately 50 min. Following electrophoresis, proteins were transferred onto polyvinylidene difluoride (PVDF) membranes (0.45 μm pore size; Millipore, Burlington, MA, USA) using a semi-wet system according to the manufacturer’s protocol (Bolt™ Transfer Buffer). The membranes were blocked in 5% non-fat dry milk (w/v) prepared in Tris-buffered saline with 0.1% Tween-20 (TBST) for 1 h at room temperature. The membranes were incubated overnight at 4 °C with primary antibodies diluted in 5% non-fat dry milk (w/v) in 0.1% TBST. Primary antibodies were against HSP60 (housekeeping), pyruvate dehydrogenase (PDH), phosphorylated PDH (phPDH) and pyruvate kinase M (PKM). All antibodies were purchased from AbCam (Cambridge, UK) and used at 1:5000. After three washes with TBST, membranes were incubated with HRP-conjugated secondary antibodies (1:20,000) for 1 h at room temperature. Signal detection was performed using an enhanced chemiluminescence (ECL) reagent (Bio-Rad, Hercules, CA, USA) and imaged with a ChemiDoc Imaging System (Bio-Rad). Densitometric analysis was performed using Bio-Rad ImageLab software, and target protein signals were normalized to the corresponding loading control (HSP60) or as a ratio of phPDH: PDH.

### PDH activity assay

PDH activity was determined using a commercially available PDH activity assay kit which was run according to the manufacturers instructions (AbCam, Cambridge, UK). Briefly, tissue was weighed and homogenized in 10x volume tris buffered saline made with 10mM NaF and 1mM PMSF (phenylmethylsulfonyl fluoride used to inhibit serine proteases). Protein was quantified using BCA assay. Protein was normalised to 2.5 mg/ml using TBS + PMSF and detergent added at 1:20. Samples were incubated with some shaking on ice for 15 min before centrifugation at 1000 g for 10 min. 150 µl per well of the precoated assay plate was added to 50 µl of PDH assay buffer and incubated overnight at 4 °C. Detection buffer was added immediately prior to running on a colourimetric plate reader running at 37 °C for 45 cycles.

### Metabolomics sample preparation

Rat plasma was used as sample matrix. For polar metabolites and lipid analysis, 40 µL of plasma was mixed with 480 µL of isotopically labelled standard mixture in MeOH: MTBE: IPA (20:15:15, v/v). Samples were vortexed for 10 min, followed by incubation at -20 °C for 45 min. Protein removal was conducted using a protein precipitation plate (55263-U, Supelco), and 100 µL of the filtrate was transferred to glass insert vials. The aliquots were evaporated and reconstituted in 50 µL water for polar metabolites analysis. The leftover filtrate was used for the lipidomics assay.

Working solutions were prepared at nine concentration levels by serial dilution from 0.125 µg/mL to 25 µg/mL in water and from 0.039 µg/mL to 10 µg/mL in chlorophorm: methanol (2:1, v/v) for polar metabolites and lipids respectively. The lipidomics master mix contained CE(18:1(9Z)), CE(18:2(9Z, 12Z)), Cer(d18:0/18:1(9Z)), Cer(d18:1/18:1(9Z)), LysoPC(18:0), LysoPC(18:1), LysoPE(18:1), PC(16:0/16:0), PC(16:0/18:1), PC(18:0/18:0), PE(16:0/18:1), TG(18:0/18:0/18:0), C16 dihydroceramide (d18:0/16:0), TG(16:0/16:0/16:0). The polar metabolites master mix contained glucose 1-phosphate, glucose 6 phosphate, fructose 6 phosphate, dihydroxyacetone phosphate, fructose 1,6 biphosphate, glyceraldehyde 3 phosphate, 2,3-diphospho-D-glyceric acid, D-2-phosphoglyceric acid, PEP/phosphpenopyruvate, lactate, glycerol-3-phosphate, pyruvate, 6-phosphogluconic acid, D-ribose 5-phosphate, acetyl coA, citrate, alpha-ketoglutarate, succinyl-CoA, succinate, fumarate, malonyl coenzyme A, malate, Itaconate, glutarate, isocitrate, oxaloacetic acid, L-arginine, glycine, alanine, valine, leucine, L-lysine, L-trytophan, L-phenylalanine, GABA, isoleucine, histidine, 3-hydroybutyric acid, L-proline, cysteinie, octanoic acid, 2-oxo-glutarate, methionine, L-threonine, L-serine, aspargine, phenylpyruvic acid, arginine, ketoleucine, 2-OH-butyric acid, taurine, omithine, ascorbic acid, creatine, xylulose-5-phosphate, erythrose-4-phosphate, sedoheptulose 7-phosphate, adenosine 5′-monophosphate.

Calibration curve samples were prepared by combining 10 µL of corresponding polar metabolites working solution*s* and 10 µL of lipid working solution, along with 240 µL of isotopically labeled internal standard mixture comprising of succinic acid-d4, valine-d8, glutamic acid-d5, glutamine-13C5, glucose-13C6, GABA-d6, glycerol-d5, indole-3-acetic acid-d5, tyrosine-d4, stearic acid d35, melatonin-d4, nicotinamide-d4, hippuric acid-d5, 18:1-d7 LYSO PE, 15:0–18:1-d7 DG, 16:0 cholesteryl-d7 ester, 18:1-d9 SM, 15:0–18:1-d7-15:0 TG, 15:0–18:1-d7-PE, 15:0–18:1-d7-PC, 18:1-d7 LYSO PC, C15 ceramide-d7, C13-dihydroceramide-d7 at 1 µg/mL concentration each in MeOH: MTBE: IPA (20:15:15, v/v).

To track analytical performance a set of solvent QC samples and pooled matrix QC samples were prepared and were injected after every 10 samples injections of the study samples. The solvent QC samples prepared out of level 6 working solution *(3.125* µg/mL for polar metabolite in the mix and 1.25 µg/mL for the lipids) according to the calibration curve samples procedure. The pooled matrix QC samples were prepared by pooling 20 µL of all samples, vortexing, and aliquoting into 40 µL portions for subsequent preparation.

### Liquid chromatography

#### Polar metabolites analysis

Liquid chromatography was performed on an ExionLC AD system, comprising two pumps, an autosampler, an AC column oven, and a system controller. A Waters Atlantis Premier BEH C18 AX column (1.7 μm, 2.1 × 100 mm) was used for separation. Mobile phases were water with 0.1% formic acid (v/v) and 10 mM ammonium formate (Phase A) and methanol with 0.1% formic acid (v/v) and 10 mM ammonium formate (Phase B). The gradient profile was as follows: 0–2 min, 100% A; 2–4 min, linear ramp to 100% B; 4–10 min, 100% B; 10.1–16 min, 100% A. The flow rate was maintained at 400 µL/min, with the column and autosampler temperatures set to 50 °C and 15 °C, respectively. Injection volume was 10 µL, and the total run time was 16 min.

#### Lipid analysis

Liquid chromatography was performed on an ACQUITY Premier UPLC system, comprising binary solvent manager, a *sample manager and* a column *manager*. A Waters ACQUITY BEH C18 column (1.7 μm, 2.1 mm × 100 mm) was used for separation. Mobile phases were 10mM of ammonium acetate in water with 0.1% formic acid (v/v) (Phase A) and 10mM of ammonium acetate in mixture acetonitrile: isopropanol (1/1, v/v) with 0.1% formic acid (v/v) (Phase B). The gradient profile was as follows: 0–2 min linear ramp 35% B to 80% B; 2–7 min, linear ramp to 100% B; 7–14 min, 100% B; 14-14.1 linear ramp to 35%B; 14.1–*21* min, *35*% B. The flow rate was maintained at 400 µL/min, with the column and autosampler temperatures set to 50 °C and 15 °C, respectively. Injection volume was 1 µL, and the total run time was *21* minutes.

#### Mass spectrometry

Broad-spectrum metabolite profiling was performed using LC–MS without pre-selection of specific analytes; however, detection was inherently constrained by extraction chemistry, chromatographic separation and mass spectrometric parameters. Lipidomic analysis was performed separately using extraction and LC–MS conditions optimised for complex lipids. Lipidomics is considered here as a subclass of metabolomics but is reported separately due to methodological differences.

#### Polar metabolites analysis

A SCIEX QTOF 6600 equipped with a Duospray ion source was employed for mass spectrometry. Ionization was performed in negative mode with the following source parameters: source temperature, 650 °C; spray voltage, -4500 V; ion source gas 1 and gas 2, 40 psi each; curtain gas, 25 psi; declustering potential (DP), -40 V; collision energy (CE), -10 V; and entrance potential (EP), -10 V. MS acquisition utilized an accumulation time of 0.4 s per scan over a mass range of 50–1000 m/z. The MS/MS data was obtained by information dependent acquisition (IDA) and was performed with a survey scan accumulation time of 0.25 s, accumulation time for MS/MS automatically selected precursor scan was set to 0.1 s, and a precursor mass tolerance of 50 ppm. Up to four MS/MS spectra were collected per cycle.

#### Lipid analysis

A SCIEX QTOF 7600 equipped with a OptiFlow (50–200µL probe) ion source was employed for mass spectrometry. Ionization was performed in positive mode with the following source parameters: source temperature, 300 °C; spray voltage, 5500 V; ion source gas 1 and gas 2, 70 psi each; curtain gas, 50 psi; declustering potential (DP), 80 V; collision energy (CE), 10 V. MS acquisition utilized an accumulation time of 0.25 s per scan over a mass range of 100–1000 m/z. The MS/MS data was obtained by information dependent acquisition (IDA) and was performed with a survey scan accumulation time of 0.1 s, accumulation time for MS/MS automatically selected precursor scan was set to 0.1 s, and a precursor mass tolerance of 50 mDa. Up to ten MS/MS spectra were collected per cycle.

### Mass spectrometry Imaging sample preparation

The Matrix-Assisted Laser Desorption/Ionization (MALDI) matrix was applied by sublimation. Super-DHB (Sigma-Aldrich) was used as a matrix at a 40 mg/mL concentration in acetone. The vacuum for the sublimation was created by MD 1 C + AK + EK chemistry vacuum system (VACUUBRAND GMBH). A volume of 750 µL was applied on the bottom of the sublimator chamber, dried with nitrogen and submitted to the vacuum with installed sample slide held above on an ice finger, the sublimation was carried out for 15 min under 140 °C.

### Mass spectrometry imaging analysis

#### Small molecules

Samples were measured on Bruker timsTOF fleX equipped with a post-ionisation laser (Bruker Daltonics) in positive mode from m/z 50 to 650 with 28 lasershots per pixel using a 20 μm laser settings (20 μm spot size; 20 μm step size). The ion mobility separation was set for 100 ms, 1/K0 was ranged from 0.4 to 1.1 V·s/cm^2^.

#### Lipids

Samples were measured on Bruker timsTOF fleX equipped with a postionisation laser (Bruker Daltonics) in positive mode from m/z 300 to 1350 with 40 lasershots per pixel using a 20 μm laser settings (20 μm spot size; 20 μm step size). The ion mobility separation was set for 200 ms, 1/K0 was ranged from 0.7 to 1.8 V·s/cm^2^.

#### Mass spectrometry data treatment

All imaging experiments were loaded into SCiLS Lab (v2023bPro, Bruker Daltonics) using the default settings with the parameters from FlexImaging. The data were root mean square (RMS) normalized, and a list of features was generated and aligned in SCiLs Lab. An ROI was defined as the striatum of the ipsilateral and contralateral hemispheres, as per the imaging protocol (Supplementary Fig. 1). For each tissue section, a receiver operating characteristic (ROC) curve was calculated to compare the ipsilateral and contralateral striatal regions. The intensities of the ion images are individually adjusted for the sections to best show the differences between hemispheres. For presentation of images, pixel intensities were RMS-normalised and expressed as a percentage of the mean normalised intensity for each metabolite across the ROI (100% = section mean). Data set was subjected to filtering. Initially, the dataset consisted of 6 control sections (from 3 individual animals) and 6 case sections (from 3 individual animals). Two samples (originating from one animal) were excluded due to brain sections being obtained from regions not representative of the striatum. Additionally, one sample (one animal) was excluded because the sectioning angle significantly deviated from the coronal plane. One sample was excluded due to noticeably lower signal intensities. After this step, 4 control samples (from 2 animals) and 4 case samples (from 3 animals) remained for further analysis.

Then the data were subjected to two distinct analytical approaches.

The ROC-based approach. To reduce false-positive feature selection, control group ROC AUC values were used to exclude features showing spurious discrimination within control samples. For each feature, the mean ROC AUC in control sections was baseline-corrected relative to 0.5 (no discrimination) and scaled to percentage units. Features with scaled control ROC values between 90% and 110% (i.e., approximately non-discriminatory in controls) were retained. This filtering ensured that selected features demonstrated hemisphere-specific discrimination in LPS-treated animals but not in control tissue.

Segmentation-based approach. A representative tissue section was selected for segmentation analysis. The selected subset was segmented using the bisecting k-means clustering algorithm with correlation distance as the similarity metric. Based on the obtained segmentation map, an ROI, predominantly localized in the ipsilateral hemisphere, was selected for further analysis (Supplementary Fig. 4). Then we assessed the spatial localization of features to this ROI within the selected tissue section. A set of 19 features showed a correlation coefficient greater than 0.2, while 29 features had a correlation coefficient lower than − 0.06. By visual inspection of the resulting ion images a feature demonstrating a reproducible signal difference between the striata of the two hemispheres within the same brain in the case group, while no such difference was observed in the control group. The feature (m/z 481.313, CCS 216.9 Å) was selected for further analysis. Co-localized features were then selected based on correlation with this feature: 47 features had a correlation coefficient greater than 0.4, and 31 features had a correlation coefficient lower than − 0.1. These lists were merged, duplicates were removed, resulting in a feature list comprising 113 unique features. The resulting feature list was further subjected to filtering based on previously established ROC values. In the case group, features with a mean ROC value within the range of 0.40–0.58 were excluded, retaining only those features demonstrating deviation from this interval. For the control group, only features with a mean ROC value within the range of 0.3–0.7 were retained. After this filtering 29 features formed the candidate list. The combined segmentation- and ROC-based feature selection strategy was designed to reduce false-positive findings and prioritise metabolites demonstrating reproducible spatial localisation across tissue sections.

### Statistics

For imaging studies, metabolic ratios were compared between hemispheres using the Wilcoxon Rank Sum test. For all other analysis standard statistical tests (ANOVA, t-test), with adjustment for normality, were performed in Prism 10. A p-value of < 0.05 was considered significant.

Lipidomics and metabolomics data were processed and statistically evaluated in R (www.r-project.org, 2025, v4.5.0) using an in-house developed script. The data were transformed by natural logarithm (ln), and the mean centering was applied. Data were evaluated using multivariate statistical analysis (principal component analysis, PCA). The univariate statistical analysis was based on the parametric t-test combined with the log2 fold-change (log2 ratio of means). P-values were adjusted for multiple testing using false discovery rate (FDR) correction and are reported in Supplementary Tables 1 & 2 together with the raw p-values. For visualization purposes, statistically significant features in the heatmap were selected based on raw p-values (p-value < 0.05). Statistically significant features (p-value < 0.05) are presented in form of a heatmap. Spearman’s R was calculated using Hmisc package and visualized as a heatmap. Functional analysis of metabolomics data was performed using Metaboanalyst v6.0 (www.metaboanalyst.ca) with mass tolerance 10 ppm.

## Results

### Intrastriatal LPS injection results in ipsilateral neuroinflammation

Our first aim was to confirm that unilateral intrastriatal LPS administration induced a localized inflammatory response. Histological analysis demonstrated increased markers of immune and vascular activation, with significant main effects of LPS and significant inflammation/hemisphere interactions across markers.

**Fig. 1:**
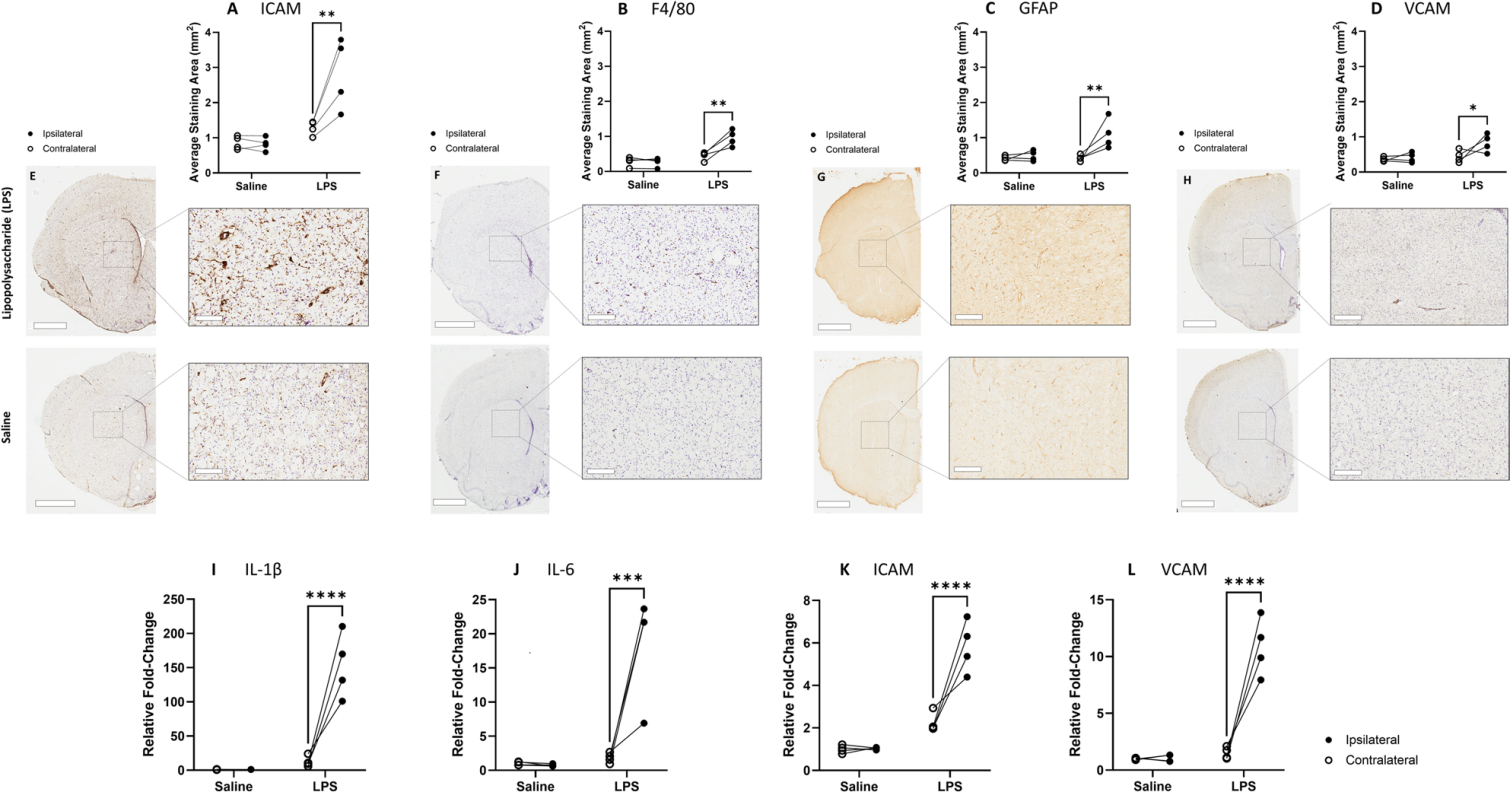
*LPS-induced neuroinflammation increases markers of immune and vascular activation in the ipsilateral hemisphere*. Representative images and quantification show increased immunoreactivity for ICAM (**A**, **E**), F4/80 (**B**, **F**), GFAP (**C**, **G**), and VCAM (**D**, **H**) in the ipsilateral hemisphere following LPS injection, indicating activation of endothelial cells, microglia/macrophages, astrocytes, and vascular endothelium respectively. Quantitative PCR demonstrated significant increases in mRNA expression of IL-1 (**I**), IL-6 (**J**), ICAM (**K**), and VCAM (**L**), with a main effect of inflammation, hemisphere, and a significant interaction between the two factors (two-way ANOVA; IL-1 and ICAM: inflammation – *p*<0.01, hemisphere – *p*<0.001, interaction – *p*<0.01; IL-6: inflammation – *p*<0.01, hemisphere – *p*<0.01, interaction – *p*<0.01; VCAM: *p*<0.001 for all effects). Post-hoc testing revealed a significant increase in gene expression in the ipsilateral hemisphere of LPS-injected animals for IL-1 (Sidak’s; *p*<0.0001; **A**), ICAM (Sidak’s; *p*<0.0001; **C**), IL-6 (Sidak’s; *p*<0.0001), and VCAM (Sidak’s; *p*<0.001). Individual data points represent biological replicates, with paired ipsilateral and contralateral measurements from each animal connected by lines; *n* = 4 per group. Scale bar on large image represents 2mm and on the inset panels, 200µm

ICAM immunoreactivity was significantly increased in the ipsilateral hemisphere of LPS-injected animals compared to contralateral tissue and controls (two-way ANOVA with Sidak’s post hoc; *p* < 0.01; Fig. 1A & E). Similarly, F4/80 expression was elevated ipsilaterally following LPS administration, consistent with increased microglial/macrophage activation (*p* < 0.01; Fig. 1B & F). GFAP staining was also increased in the ipsilateral hemisphere, indicating astrocyte activation (*p* < 0.01; Fig. 1C & G). VCAM expression showed a more modest but still significant ipsilateral increase following LPS injection (*p* < 0.05; Fig. 1D & H).

Consistent with the histological findings, inflammatory gene expression analysis revealed robust ipsilateral upregulation of pro-inflammatory mediators. IL-1β and ICAM mRNA showed significant main effects of inflammation and hemisphere, as well as significant interaction effects (two-way ANOVA; *p* < 0.01 for all; Fig. 1I & K), with post hoc analysis confirming marked increases in the ipsilateral hemisphere following LPS injection (*p* < 0.0001). IL-6 expression demonstrated a similar pattern, with significant main and interaction effects (*p* < 0.01), driven by increased ipsilateral expression in LPS-treated animals (*p* < 0.0001; Fig. 1J). VCAM mRNA levels were likewise significantly elevated in the ipsilateral hemisphere after LPS administration (*p* < 0.001; Fig. 1L).

Together, these findings confirm that the unilateral LPS injection produced a robust and spatially localized neuroinflammatory response at 24 h.

### Acute neuroinflammation results in decreased bicarbonate: pyruvate and bicarbonate: lactate in the absence of structural changes

**Fig. 2:**
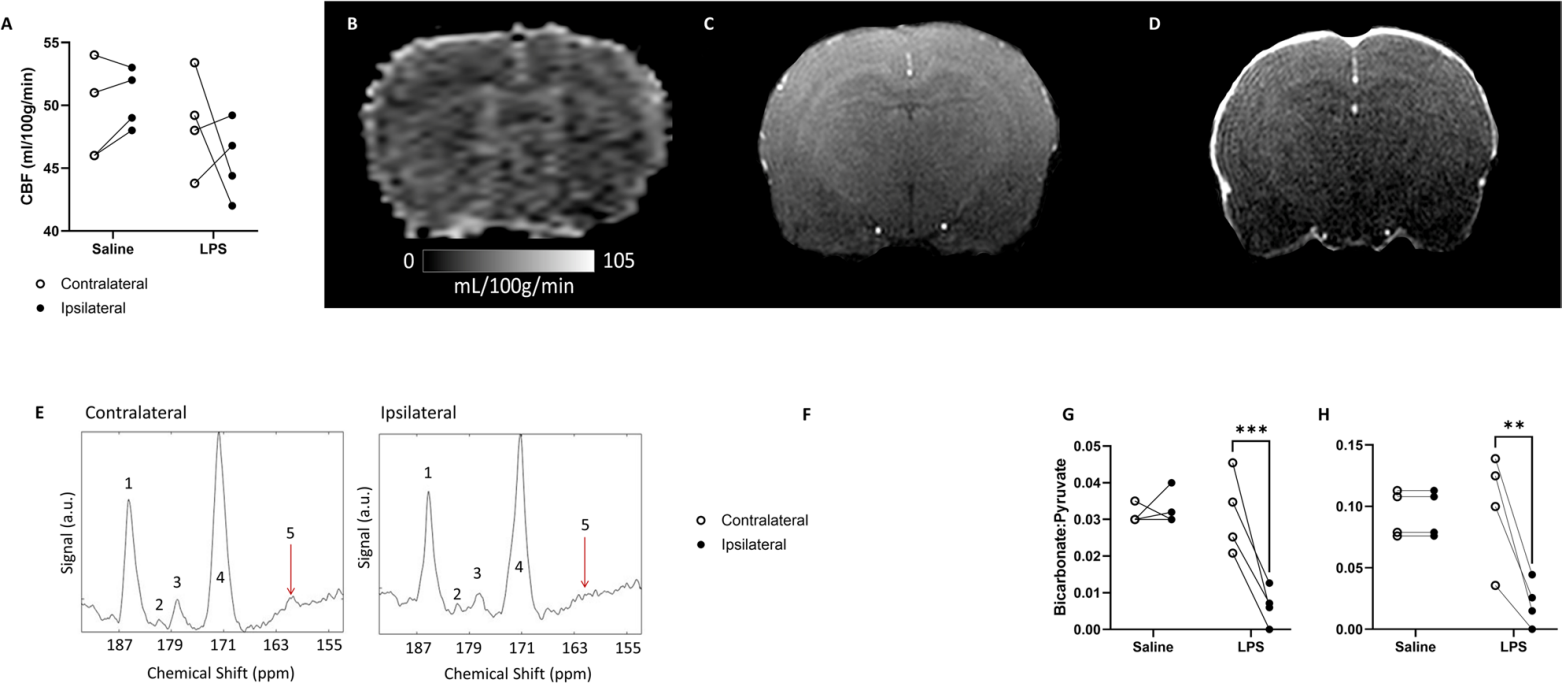
*Assessment of cerebral perfusion and pyruvate metabolism following LPS injection*. There was no significant difference in cerebral blood flow between groups (**A**; t-test; *p* = 0.6), with an example CBF map from an LPS brain shown in **B**. Blood-brain barrier integrity as assessed using pre- and post- injection of gadolinium contrast (**C** and **D**, respectively) showed no discernible contrast agent extravasation. Example hyperpolarized spectra from the ipsilateral hemisphere in the LPS brain showed a decrease in ^13^C bicarbonate formation compared to contralateral (E) - from left to right: (1) lactate ~185 ppm, (2) pyruvate-hydrate ~181 ppm, (3) alanine ~178ppm, (4) pyruvate ~171 ppm, (5) bicarbonate ~161 ppm (see Supplementary Figure 2 for labelled peaks). No significant difference in [1-13C] lactate: [1-13C] pyruvate was observed (**F**; *p* > 0.86), however a significant main effect of inflammation (p < 0.05), hemisphere (*p* < 0.01), and interaction (*p* < 0.01) was observed for the ^13^C bicarbonate: [1-13C] pyruvate ratio (**G**). For the ^13^C bicarbonate: [1-13C] lactate ratio, there was a main effect of hemisphere (*p* < 0.05) and interaction (*p* < 0.01), but not of inflammation (*p* = 0.06) (**H**). Post-hoc testing revealed significant differences between contralateral and ipsilateral hemispheres in LPS-injected animals (Sidak’s; **G**: *p* < 0.001; **H**: *p* < 0.01). Individual data points represent biological replicates, with paired ipsilateral and contralateral measurements from each animal connected by lines; *n* = 4 per group

Having established that unilateral LPS administration induced a local inflammatory response, we next assessed whether this was accompanied by overt structural brain injury. Using perfusion weighted imaging and gadolinium we determined there was no significant difference in cerebral blood flow after an intrastriatal injection of LPS (t-test: *p* = 0.6; Fig. 2A & B. More specifically, the observed difference in CBF between groups was within physiological variability (considered at < 10% change; there was a ~ 5–6% reduction in LPS-treated animals), and the 90% confidence interval (− 9.6% to − 1.5%) fell within a predefined ± 10% range considered to represent biologically subtle changes. In addition there was no significant blood brain barrier breakdown (Fig. 2C & D; pre- and post-gadolinium, respectively). These findings are consistent with the low-dose, focal inflammatory model employed here, which is designed to elicit neuroinflammation without producing infarction or gross tissue loss.

We therefore used hyperpolarized [1-¹³C] MRI to determine whether metabolic alterations could be detected in the absence of overt structural damage. Following injection of [1-¹³C]pyruvate, metabolite ratios were quantified separately in the ipsilateral and contralateral hemispheres (Fig. 2E, Supplementary Fig. 2).

No significant differences were observed in the [1-¹³C]lactate: [1-¹³C]pyruvate ratio, with no main effect of inflammation, no main effect of hemisphere, and no interaction between factors (two-way ANOVA; inflammation *p* = 0.86; hemisphere *p* = 0.99; interaction *p* = 0.99; Fig. 2F).

In contrast, bicarbonate-related metrics were altered. The ¹³C-bicarbonate: [1-¹³C]pyruvate ratio showed a significant main effect of inflammation (*p* < 0.05), a significant main effect of hemisphere (*p* < 0.01), and a significant inflammation × hemisphere interaction (*p* < 0.01; Fig. 2G). Post hoc testing revealed a significant reduction in the bicarbonate: pyruvate ratio in the ipsilateral hemisphere of LPS-treated animals compared to the contralateral hemisphere (Sidak’s *p* < 0.001).

Similarly, the ¹³C-bicarbonate: [1-¹³C]lactate ratio demonstrated a significant main effect of hemisphere (*p* < 0.05) and a significant interaction between inflammation and hemisphere (*p* < 0.01), although the main effect of inflammation did not reach significance (*p* = 0.06; Fig. 2H). Post hoc analysis again identified a significant reduction in the ipsilateral hemisphere of LPS-injected animals (Sidak’s *p* < 0.01).

Together, these findings indicate that acute focal neuroinflammation is associated with reduced bicarbonate production relative to pyruvate and lactate in the ipsilateral hemisphere, despite preserved cerebral blood flow and absence of overt structural damage.

### Acute neuroinflammation changes the peripheral metabolite profile

**Fig. 3:**
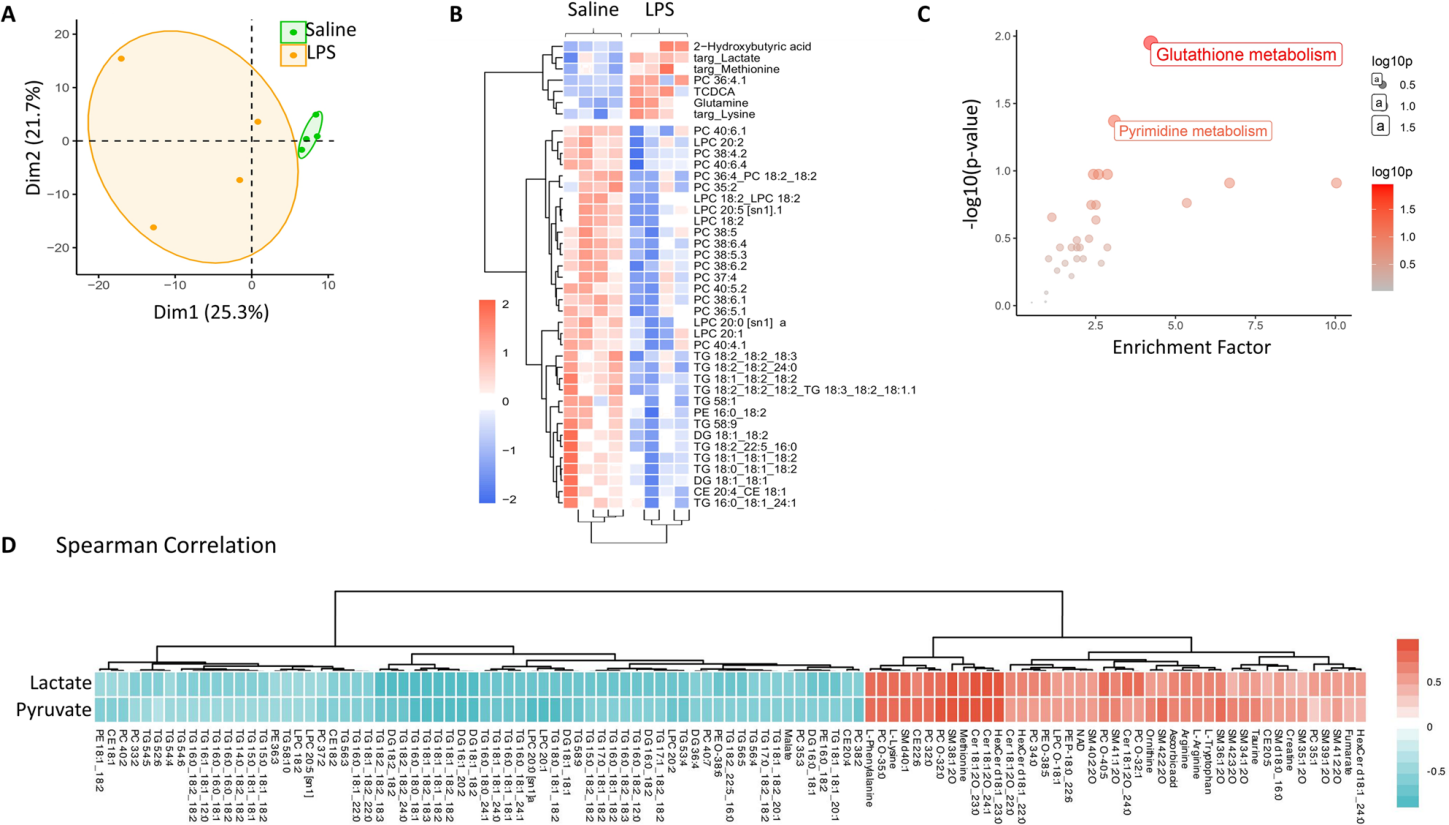
*Plasma metabolome alterations are dominated by elevated glycerophospholipids and triacylglycerols*. **A** Principal component analysis (PCA) scores plot of the plasma metabolome and lipidome, showing separation between LPS-treated and saline control animals (PC1 and PC2 explain 25.3% and 21.7% of the variance, respectively). **B** Heatmap of significantly altered metabolites and lipids between LPS and saline groups (Student’s t-test; FDR-adjusted p-values reported in Supplementary Tables 1 & 2). The colour scale represents row-wise z-score normalised metabolite abundances (z = (x − mean) / SD), with blue indicating lower and red indicating higher relative abundance (range −2 to +2). **C** Functional pathway enrichment analysis performed in MetaboAnalyst using significantly altered plasma metabolites (m/z features) with associated p-values. Dot colour and size both represent −log10(p-value) from pathway enrichment analysis, such that larger and more intensely coloured (red) circles indicate greater statistical significance (lower *p*-values). **D** Spearman correlation heatmap (|R| > 0.5) illustrating metabolites associated with circulating plasma lactate and pyruvate levels. The colour scale represents Spearman correlation coefficients (ρ), ranging from −1 (blue; strong negative correlation) to +1 (red; strong positive correlation)

We next profiled plasma metabolites and lipids to determine whether systemic metabolic changes accompanied the central inflammatory response. Multivariate analysis revealed clear separation between LPS-treated and control animals, as visualized by principal component analysis (PCA; Fig. 3A), supporting a global shift in the plasma metabolome and lipidome.

Consistent with this global separation, numerous plasma metabolites differed significantly between groups (Fig. 3B; Supplementary Fig. 3, Supplementary Tables 1 & 2), including several phospholipids, diacylglycerols, and other lipid classes.

Pathway enrichment analysis of significantly altered metabolites indicated involvement of glutathione metabolism and pyrimidine metabolism in the systemic response to LPS administration (Fig. 3C).

Correlation analysis further revealed multiple lipid and metabolite species associated with circulating plasma lactate and pyruvate levels (Fig. 3D; Supplementary Tables 1 & 2), indicating coordinated relationships between glycolytic intermediates and broader metabolic alterations.

Together, these findings suggest that focal LPS-induced neuroinflammation is associated with systemic metabolic alterations in plasma, although the relative contribution of central versus peripheral inflammatory drivers cannot be determined from the present data.

### Acute neuroinflammation changes metabolism in the ipsilateral hemisphere

Spatial metabolomics was performed to identify region-specific metabolic alterations associated with focal neuroinflammation. This approach enables anatomical localization of metabolite changes within discrete brain regions and complements the *in vivo* imaging findings.

Comparison of ROIs drawn in the ipsilateral and contralateral hemispheres (Supplemental Fig. 1) revealed multiple features exhibiting lateralized differences in LPS-treated animals. The four highest-ranked metabolites based on left–right differential intensity were cyclidine 2′,3′-cyclic phosphate (Supplemental Fig. 5A–C), glutathione (Supplemental Fig. 5D–F), N-palmitoyl phenylalanine (Supplemental Fig. 5G–I), and prostaglandin species (Supplemental Fig. 5J–L). Representative images demonstrate increased signal intensity for these metabolites in the ipsilateral striatum of LPS-injected animals relative to the contralateral hemisphere and vehicle-treated controls.

To assess discriminatory performance, receiver operating characteristic (ROC) analyses were conducted for each metabolite comparing ipsilateral versus contralateral signal. In LPS-treated animals, ROC values ranged from 0.624 to 0.650, whereas control animals demonstrated values close to chance (0.459–0.531), indicating that lateralized metabolic differences were specific to the inflammatory condition (Supplementary Table 3).

**Fig. 4:**
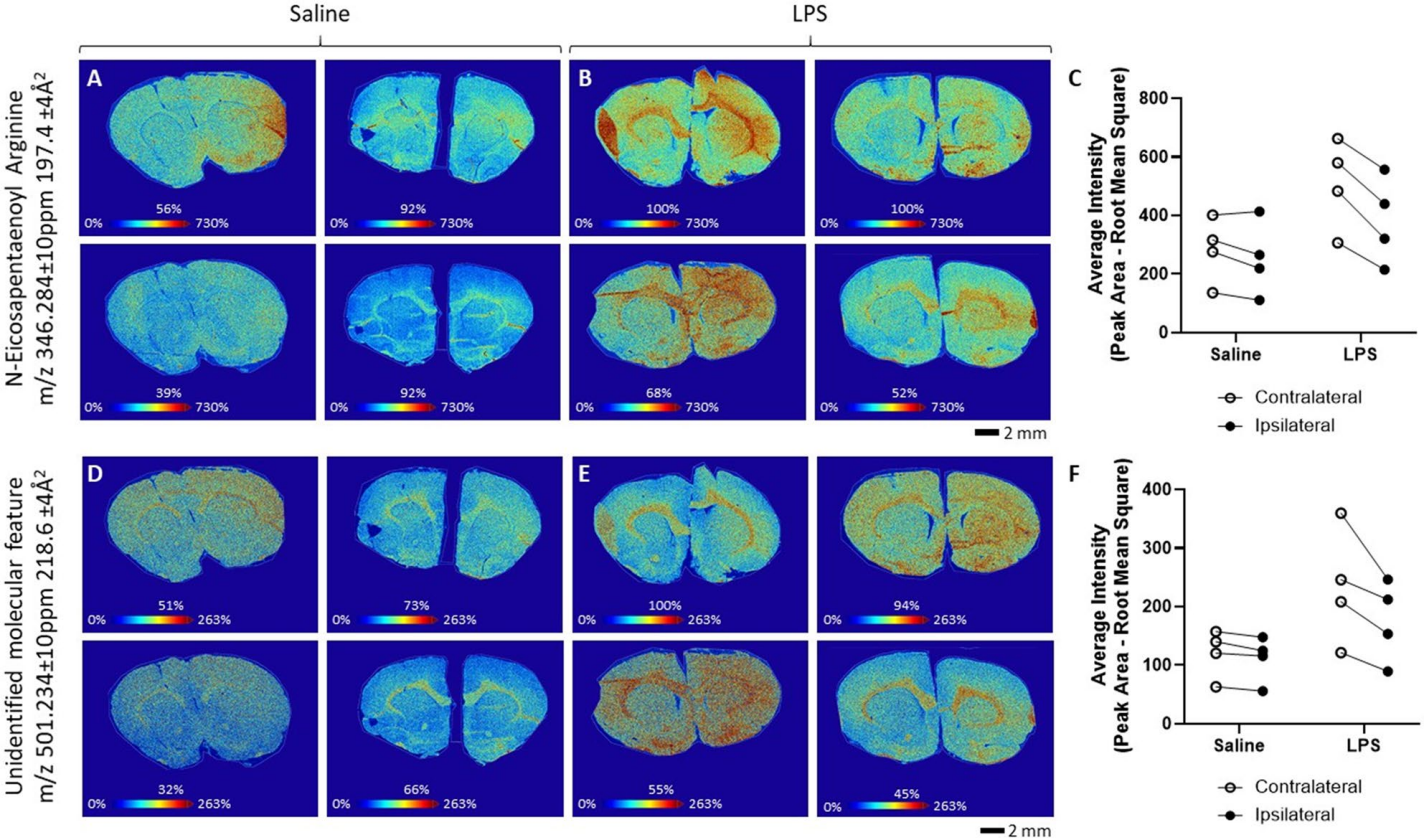
*LPS-induced neuroinflammation results in ipsilateral changes in selected metabolites*. Representative images are high spatial resolution (20µm) root mean square normalized ion images of LPS and saline injected brains for N-Eicosapentaenoyl Arginine (**A** & **B**), unidentified molecular feature (**D** & **E**). Pixel intensities are RMS-normalised and expressed relative to the mean intensity within each section (100% = section mean); colour scales therefore reflect relative spatial distribution and are independently scaled per section. Graphs show average distribution of intensities of selected peak after root mean square normalization between the striata of selected samples (defined ROI) for N-Eicosapentaenoyl Arginine (**C**), unidentified molecular feature (**F**). Individual data points represent biological replicates, with paired ipsilateral and contralateral measurements from each animal connected by lines; *n* = 4 per group; scale bar on representative images shows 2 mm

We also performed cluster analysis of the polar metabolites, and this resulted in a clear cluster in the injected striatum (Supplementary Fig. 4B, blue cluster). When we compared these metabolites across all the brain sections there were left right differences observed in several metabolites consistently (Supplementary Table 5). The two most consistent metabolites that showed left right differences were tentatively identified as N-eicosapentaenoyl arginine and unidentified molecular feature (Fig. 4A-B). These were downregulated in the ipsilateral hemisphere of the LPS injected animals (Fig. 4E). These did not show an up regulation in the saline brains (Fig. 4C-D, E).

Together, these findings suggest lateralized metabolic alterations in the ipsilateral striatum following focal LPS-induced neuroinflammation, although confirmation in larger cohorts will be required to establish robustness.

### Changes in energy metabolism measurements are accompanied by a trend in changes in pyruvate dehydrogenase activity

To determine whether the observed changes in pyruvate metabolism were associated with altered regulation of pyruvate dehydrogenase (PDH), we assessed expression of pyruvate dehydrogenase kinase (PDK) isoforms, PDH phosphorylation status, and enzymatic activity.

**Fig. 5:**
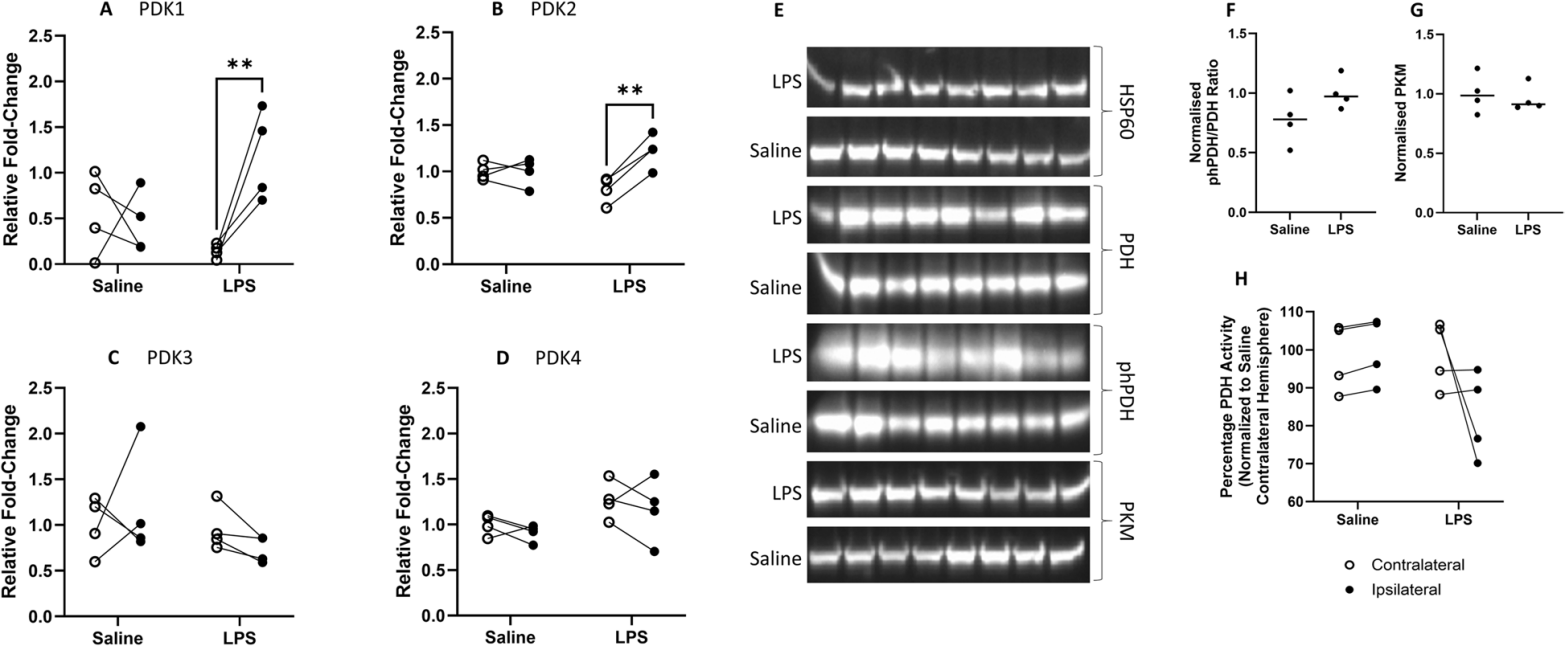
*Inflammation-associated changes in pyruvate metabolism are accompanied by altered PDK1 and 2 mRNA expression and trends in PDH regulation*. mRNA expression of PDK isoforms was measured in ipsilateral and contralateral brain hemispheres. Significant changes were observed in PDK1 (**A**) and PDK2 (**B**) but not PDK3 (**C**) or PDK4 (**D**). Both PDK1 and PDK2 mRNA expression showed a significant main effect of inflammation (two-way ANOVA; *p* < 0.0001) with increased expression in the ipsilateral hemisphere of LPS-injected animals (Sidak’s; *p* < 0.01; **A** & **B**). Western blotting revealed a non-significant trend toward increased phPDH:PDH ratio in LPS-injected animals (**E**, **F**; *p* = 0.06). PKM expression was unchanged between groups (**G**). PDH activity assay showed a trend toward reduced enzymatic activity in the ipsilateral hemisphere following LPS injection (**H**; *p* = 0.06). Individual data points represent biological replicates, with paired ipsilateral and contralateral measurements from each animal connected by lines; *n* = 4 per group.

PDK1 and PDK2 mRNA expression demonstrated a significant main effect of inflammation (two-way ANOVA; *p* < 0.0001 for both; Fig. 5A & B), with post hoc analysis confirming increased expression in the ipsilateral hemisphere following LPS injection (Sidak’s *p* < 0.0001). No significant main effects or interactions were observed for PDK3 or PDK4 expression (Fig. 5C & D).

Western blot analysis revealed an increase in the phospho-PDH: total PDH ratio in the ipsilateral hemisphere of LPS-injected animals; however, this did not reach statistical significance (*p* = 0.06; Fig. 5E & F). Consistent with this pattern, PDH activity assays showed a relative reduction in enzymatic activity in the ipsilateral hemisphere following LPS administration, which similarly did not reach statistical significance (*p* = 0.06; Fig. 5H).

To assess whether these findings reflected broader dysregulation of glycolytic enzymes, PKM expression was also measured and showed no significant differences between groups (Fig. 5G).

Together, these findings suggest that focal neuroinflammation is associated with upregulation of PDK1/2 and a trend toward reduced PDH activity in the ipsilateral hemisphere, consistent with altered oxidative metabolism.

## Discussion

This study integrated hyperpolarized ^13^C MRI, spatial metabolomics, peripheral metabolomics, and enzymatic analyses to characterise metabolic alterations following an acute focal LPS challenge in the brain. As a feasibility and proof-of-concept investigation with a small cohort, our objective was not to establish definitive mechanistic causality but to determine whether these complementary approaches could detect early metabolic changes associated with neuroinflammation. In addition to hemisphere-based comparisons, we implemented a segmentation-based spatial metabolomics approach to identify metabolically distinct regions in an unbiased manner. This enables the detection of localized metabolic patterns that are not constrained by predefined anatomical boundaries and provides a more nuanced view of region-specific metabolic responses to inflammation.

The model produced a robust ipsilateral inflammatory response, confirmed by histological and molecular markers of vascular activation, microglial and astrocytic reactivity, and cytokine upregulation. Notably, these inflammatory changes occurred in the absence of overt structural injury or macroscopic BBB disruption, providing a controlled context in which to assess metabolic alterations.

Although our LPS dose was low (10 ng), contralateral effects are not unexpected in central inflammatory models. Higher-dose unilateral LPS injections have demonstrated bilateral histopathological changes [24], supporting the capacity for inflammatory signalling to propagate beyond the initial site of insult. Our work used a dose 500x less than this and was designed to induce mild and relatively local inflammation, which is supported by our histological findings.

Whilst the central injection of LPS has often been considered a reductionist way of studying neuroinflammation, it is being increasingly used as a way of investigating how neuroinflammation might precipitate diseases such as Alzheimer’s and Parkinson’s [25]. However, in the context of a pilot-style study such as ours, this reductionism is an advantage, allowing us to isolate early metabolic effects with fewer confounding variables. In fact, we know that inflammation often precedes physical and cognitive impairment in a variety of neurodegenerative diseases [26, 27]. Novel therapies targeting inflammation [28] and metabolic processes [29] are becoming increasingly in vogue and, as such, it will become ever more important to be able to monitor their efficacy in ongoing trials. Conventional measures of therapeutic efficacy often rely upon measures of disability or cognition, or on imaging parameters such as atrophy, all of which may take several years to manifest [30]. Changes in inflammation, or in metabolic processes within cells, will occur on a much more rapid time-scale and as such will be better biomarkers for clinical trials of novel therapies targeting these processes [31]. Despite this, the technology to effectively monitor inflammation, especially within closed compartments such as the CNS, remains limited.

To provide the steps needed for the development of a clinical biomarker of early inflammatory activity, we require novel, safe, technologies capable of imaging longitudinal inflammation, a dynamic and multi-cellular process. Here, combined destructive (MALDI, histopathology, molecular biology) and translational (hyperpolarized MRI, plasma metabolomics) technologies to were used to readout changes in metabolism related to acute inflammatory challenge. Although our data are preliminary, these findings provide early proof-of-concept that hyperpolarized ^13^C approaches may contribute to such a biomarker pipeline [32]. Understanding how the brain generates energy generally, not just in the context of neuroinflammation, is crucial given the number of diseases where metabolic disruption is an underlying pathological hallmark. Metabolism can be broadly divided into biosynthetic processes, which produce intermediates used to build cellular components, and energy-generating processes, which break down nutrients like glucose to produce ATP.

In addition to the metabolic alterations detected by hyperpolarized spectroscopy of the CNS, results demonstrated clear evidence of systemic metabolic reprogramming following acute neuroinflammatory challenge. Plasma metabolomics and lipidomics revealed a distinct separation between LPS-treated and control animals and identified significant shifts in metabolite and lipid abundance. The correlations between circulating lactate/pyruvate and specific plasma metabolites and lipid species further suggest that CNS inflammation induces a coordinated metabolic response across central and peripheral compartments. This systemic profile is consistent with established metabolic responses to innate immune activation, where inflammatory signalling drives increased glycolytic flux, altered TCA-cycle activity, and enhanced lipid mediator synthesis [31, 33]. The plasma mass-spectrometry data therefore provide an external, easily accessible signature of the metabolic state induced by neuroinflammation and serve to validate the shifts in CNS pyruvate metabolism detected by hyperpolarized MRI. These observations should be interpreted cautiously given the limited sample size, but they reinforce the central aim of this feasibility study: to show that multimodal metabolic profiling can reveal coordinated CNS–peripheral responses to acute inflammation.

Several groups have reported systemic metabolomic alterations in models of neuroinflammation and neurodegeneration, including shifts in kynurenine pathway metabolites, glutathione metabolism, and lipid mediator profiles following central [34, 35] or systemic LPS exposure [36–38]. These findings are consistent with our observation of altered glutathione and pyrimidine metabolism and support the concept that inflammatory signalling induces coordinated metabolic reprogramming across tissues. However, unlike protein biomarkers such as GFAP, which primarily reflect astrocytic injury or reactivity [39, 40], plasma metabolite profiles capture pathway-level changes in cellular bioenergetics and redox state [41, 42]. These modalities should therefore be considered complementary rather than competitive, with protein markers providing cell-type specificity and metabolomics reflecting broader metabolic state.

Importantly, the observation that peripheral metabolic alterations occur alongside early CNS metabolic changes suggests that hyperpolarized MRI is capturing biologically relevant metabolic reprogramming rather than solely non-specific changes in perfusion or tissue integrity. Although subtle perfusion alterations cannot be excluded due to limited power, the absence of a significant CBF increase suggests that the observed metabolic changes are unlikely to be secondary to gross perfusion alterations. However, plasma metabolite signatures should not be assumed to arise directly from brain inflammation. Systemic LPS exposure is known to induce widespread immune and metabolic responses, and circulating metabolites likely reflect integrated whole-body effects rather than brain-specific processes. As systemic inflammatory mediators (e.g., circulating cytokines or immune cell populations) were not assessed in this cohort, the relative contribution of central versus peripheral drivers cannot be determined.

It is also important to acknowledge that altered oxidative metabolism and lactate dynamics are not unique to neuroinflammation. Mitochondrial dysfunction and shifts in glycolytic–oxidative balance are observed across a range of neurological conditions, including ischaemia, neurodegeneration, and traumatic brain injury. Accordingly, hyperpolarized ^13^C MRI is unlikely to function as a disease-specific marker in isolation. Rather, its strength lies in providing dynamic, regionally resolved information about metabolic flux that can complement structural imaging, protein biomarkers, and clinical phenotyping. In this context, among the spatially localized metabolic features observed in the brain, one metabolite tentatively identified as an N-acyl amino acid (N-eicosapentaenoyl arginine) was reduced in the ipsilateral hemisphere. N-acyl amino acids are increasingly recognised as bioactive lipid mediators involved in metabolic and inflammatory signalling, although the specific functional role of this species in the brain remains unclear. Its altered distribution may reflect localized changes in lipid-associated inflammatory or metabolic pathways, but further structural confirmation and functional studies will be required to clarify its biological significance.

Nevertheless, the parallel detection of central metabolic imaging changes and peripheral metabolic alterations raises the possibility that combined metabolomics and metabolic imaging approaches may provide a complementary multimodal biomarker framework for monitoring inflammatory activity *in vivo* [43]. Future studies with larger cohorts, integrated systemic immune profiling, and models incorporating mixed pathology such as ischemia or multiple sclerosis will be essential to validate and refine these early observations.

Beyond the basic metabolic profiles, results demonstrated that there were changes in one of the key enzymes that regulate entry of pyruvate into the TCA cycle, specifically in pyruvate dehydrogenase, with corresponding changes in mRNA expression levels of its regulators, pyruvate dehydrogenase kinases 1 and 2. These data, showing metabolic changes in the brain in response to LPS, are complementary to a previous study which utilised hyperpolarized MRI to assess chronic changes in brain metabolism where the authors showed an increase in [1-^13^ C]lactate production in the brain at day 7 [11]. This increase correlated with elevated Iba-1 and GFAP expression, and the authors postulate that the increase in lactate comes from the increased number of astrocytes and microglia. However, Le Page et al. did not aim to detect ^13^C bicarbonate as a co-polarized solution of [1-^13^ C]pyruvate and ^13^C urea was infused, which may have obscured the ^13^C bicarbonate peak. Here we have demonstrated, at an earlier time point, that changes in ^13^C bicarbonate due to LPS challenge are detectable just 24 h after insult, before meaningful changes in lactate formation are observed. These results thus provide early evidence that mitochondrial dysfunction may precede glycolytic shifts during acute neuroinflammation, offering new insight into the temporal sequence of metabolic changes in the brain.

Despite the fact that we are coming up to 90 years since the discovery of the citric acid cycle, technological developments have continually advanced our sensitivity to detect metabolic intermediates *in vivo* in the brain [44]. Our clinical capacity to rapidly image cellular processes is currently limited. Whilst MR spectroscopy can be used to measure specific cellular markers, such as glutamate or creatine, the ability to track dynamic changes in metabolism is often challenging and metabolites are required to be in the millimolar range to be resolved. Imaging inflammation using PET is feasible and many ligands show significant changes in pathology. However, the repeated exposure to radiation means this technology is not best suited to longitudinal studies in chronic disease, such as those that might be required to study the development of post-stroke dementia, for example. By using hyperpolarised ^13^C labelled metabolites that readout on the TCA cycle, we have more of a handle on the downstream processes occurring within the CNS in response to injury and disease [6, 8, 11].

Whilst using hyperpolarized MRI as a tool for early detection of inflammatory activity may not currently be practical in the clinical setting, due to the often-late presentation of patients, it does point to the ability of the technology to provide a direct readout of druggable targets in the pre-clinical development and testing pipeline. To detect chronic immune activity in the brain, through the increased production of lactate, may be a more realistic use of the technology in neuroinflammatory disease settings where there are, otherwise, a lack of structural or functional changes the brain. By using static and dynamic means of measuring metabolism we have demonstrated that inflammation affects apparent CNS metabolism, as well as demonstrating the feasibility of using changes in metabolism as a proxy for measuring neuroinflammatory processes in the brain.

## Limitations and conclusion

A major limitation of this study is the small cohort size (*n* = 4 per group), which reduces statistical power and increases uncertainty around interaction effects. The study was designed as a feasibility experiment to detect large metabolic shifts and establish methodological integration across imaging and metabolomics platforms. Accordingly, findings should be interpreted as preliminary and hypothesis-generating. Larger, adequately powered studies will be required to establish reproducibility and to refine mechanistic interpretation and to develop the use of hyperpolarised imaging.

Most notably, the spatial metabolomics analysis did not reveal uniform hemispheric shifts but instead identified heterogeneous, feature-dependent changes, with some metabolites showing localized alterations while others remained unchanged. Such heterogeneity is consistent with the spatially complex nature of neuroinflammatory responses, which involve multiple cell types and microenvironmental factors rather than a uniform tissue-wide effect. Accordingly, the observed differences are better interpreted as region-specific and spatially localized metabolic alterations rather than consistent hemispheric lateralization.

The rodent model of neuroinflammation is somewhat reductionist and does not necessarily recapitulate the complexities of human disease. However, we have demonstrated that metabolic imaging in particular is translatable in a model of stroke [32] and our ongoing research using chronic models of stroke and multiple sclerosis aim to introduce additional levels of pathological complexity.

A more specific limitation in this study relates to tissue handling following extraction. While cold saline perfusion and rapid processing were employed, metabolic activity is not immediately quenched post-mortem. As such, metabolite levels may be influenced not only by degradation but also by post-mortem increases in certain metabolites due to ongoing enzymatic activity. Although comparison of ipsilateral and contralateral hemispheres helps to mitigate some variability, it does not fully exclude the possibility that differences in post-mortem metabolic dynamics between conditions may contribute to the observed contrasts. Nevertheless, the spatial consistency of selected metabolic features within defined regions supports the interpretation that at least part of the observed signal reflects biologically relevant responses to inflammation rather than solely post-mortem artefacts.

A limitation of the present study is that BBB integrity was assessed only qualitatively using post-gadolinium T1-weighted imaging. While no macroscopic contrast enhancement was observed, this approach is insensitive to subtle permeability changes. More sensitive quantitative imaging (e.g., dynamic contrast-enhanced MRI), tracer-based assays (e.g., Evans Blue, radiolabelled sucrose/albumin), or histological assessments of tight junction proteins would be required to exclude low-level BBB disruption. As such, subtle barrier alterations cannot be definitively ruled out.

Our main aim with this preliminary work was to demonstrate the feasibility of imaging the brain using hyperpolarized pyruvate after an inflammatory challenge and to provide evidence that this signal (supported by complementary spatial metabolomics and molecular biology ex vivo) can be used as a proxy for imaging ongoing inflammation *in vivo*. Our data builds on previous published work [11] and confirms hyperpolarized metabolic imaging is an effective way to monitor CNS inflammation.

## Supplementary Information

Supplementary Material 1: Fig. S1. *Illustration of ROI used for imaging and spatial metabolomics*. Rat brain atlas image (Gaida.ca) shows the coronal slice at +1.5mm from Bregma with the ipsilateral (red) and contralateral (green) striatum outlined. These areas were used as ROI for imaging analysis (spectra and CBF measurements) as well as for spatial metabolomics. An example of extracted spatial metabolomics data is inset here.

Supplementary Material 2: Fig. S2. *Hyperpolarized [1-¹³C]pyruvate spectra from saline-treated control animals*. Representative hyperpolarized ¹³C spectra acquired from the ipsilateral and contralateral hemispheres of saline-injected animals. Metabolite resonances are labelled (lactate ~185 ppm, pyruvate-hydrate ~181 ppm, alanine ~178 ppm, pyruvate ~171 ppm, bicarbonate ~161 ppm). In contrast to LPS-treated animals (Figure 2E–F), no visible difference in the bicarbonate peak (indicated by red arrow) is observed between hemispheres in control brains.

Supplementary Material 3: Fig. S3. Volcano plot of plasma metabolomics data comparing LPS-treated and saline control animals. Volcano plot showing differential plasma metabolites between LPS and saline groups. The x-axis represents log₂ fold-change (LPS vs. saline), and the y-axis represents −log₁₀(p-value). Each point corresponds to a detected metabolite feature (m/z value). Features meeting statistical significance after false discovery rate (FDR) correction are highlighted. Full statistical details, including raw p-values, FDR-adjusted p-values, and fold-changes, are provided in Supplementary Tables 1 and 2.

Supplementary Material 4: Fig. S4. Segmentation demonstrates that inflammation induces hemisphere-wise changes in the rat brain. A representative tissue section was selected and subjected to segmentation using the bisecting k-means clustering algorithm, with correlation distance employed as the similarity metric. A - Segmentation map showing the resulting clusters in a representative inflammation-induced tissue section. B - The same segmentation map with the segment of interest highlighted. This segment was further used as an ROI for subsequent co-localization analysis of features relative to the ROI.

Supplementary Material 5: Fig. S5. LPS-induced neuroinflammation results in ipsilateral changes in selected metabolites. Representative images are high spatial resolution (20µm) root mean square normalized ion images of LPS and saline injected brains for cytidine-2’3-cyclic phosphate (A & B), glutathione (D & E), N-palmitoyl phenylalanine (G & H) and prostaglandin (J & K). The brightness scale is adjusted sample-wise to assess sample to sample intensity variations. Graphs show average distribution of intensities of selected peak after root mean square normalization within the striatum (defined ROI) for cytidine-2’3-cyclic phosphate (C), glutathione (F), N-palmitoyl phenylalanine (I) and prostaglandin (L). Individual data points are shown, with the group mean indicated by a horizontal line. Colour scale represents relative pixel intensity expressed as a percentage of the mean RMS-normalised intensity for that metabolite across the ROI (100% = section mean). Scale bar on representative images shows 800µm.

Supplementary Material 6.

Supplementary Material 7.

Supplementary Material 8.

Supplementary Material 9.

Supplementary Material 10.

## Data Availability

The datasets used and/or analysed during the current study are available from the corresponding author on reasonable request.
