# Supplementary figures and images for: Feasibility of multimodal metabolic analysis for detecting early changes in acute neuroinflammation
Source: J Neuroinflammation. 2026 May 4;23:228. doi: 10.1186/s12974-026-03839-7 (PMC13335137; doi:10.1186/s12974-026-03839-7)

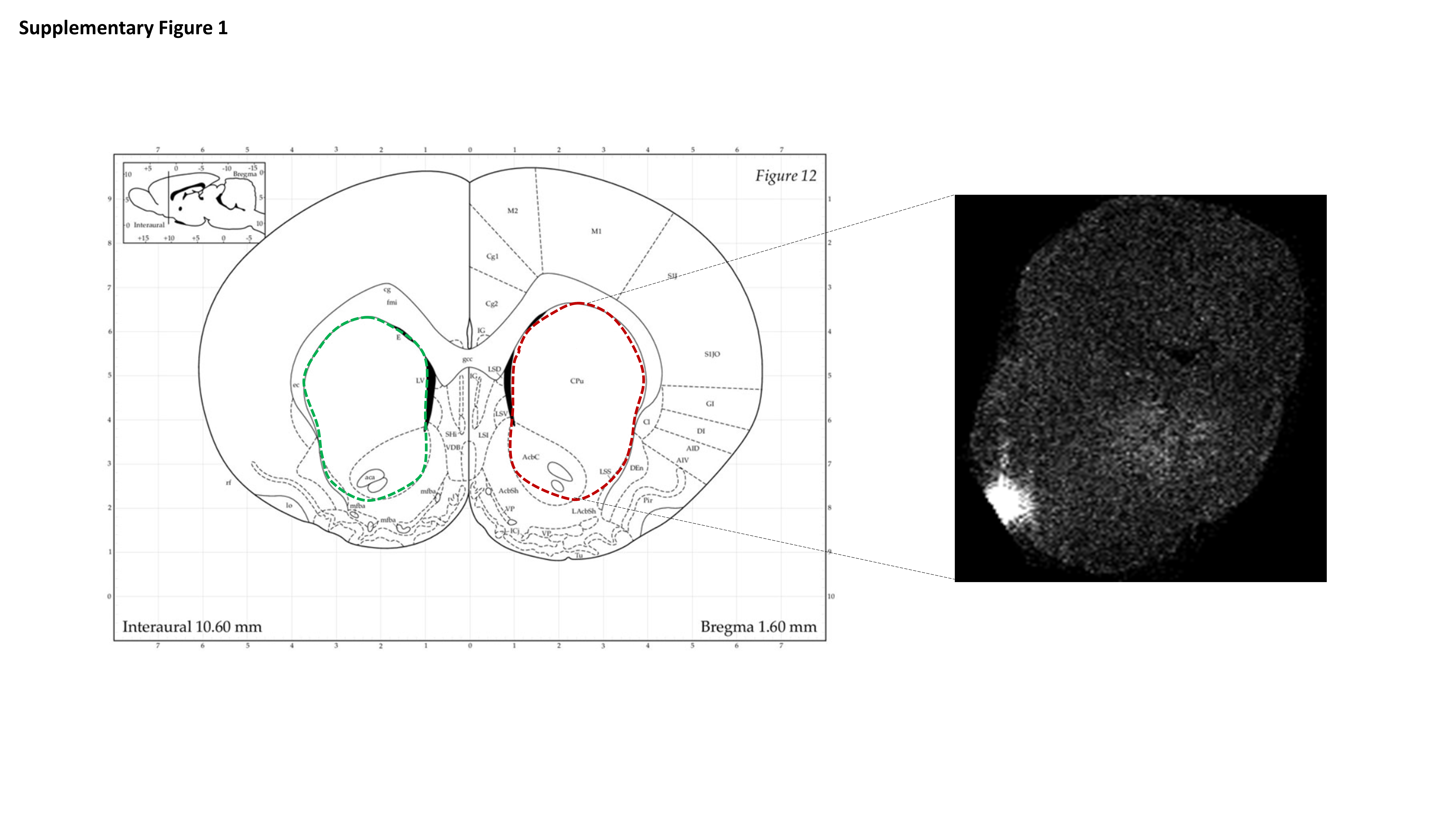

Supplement: Supplementary file 1 — Supplementary Material 1: Fig. S1. Illustration of ROI used for imaging and spatial metabolomics. Rat brain atlas image (Gaida.ca) shows the coronal slice at +1.5mm from Bregma with the ipsilateral (red) and contralateral (green) striatum outlined. These areas were used as ROI for imaging analysis (spectra and CBF measurements) as well as for spatial metabolomics. An example of extracted spatial metabolomics data is inset here. [file 12974_2026_3839_MOESM1_ESM.png]

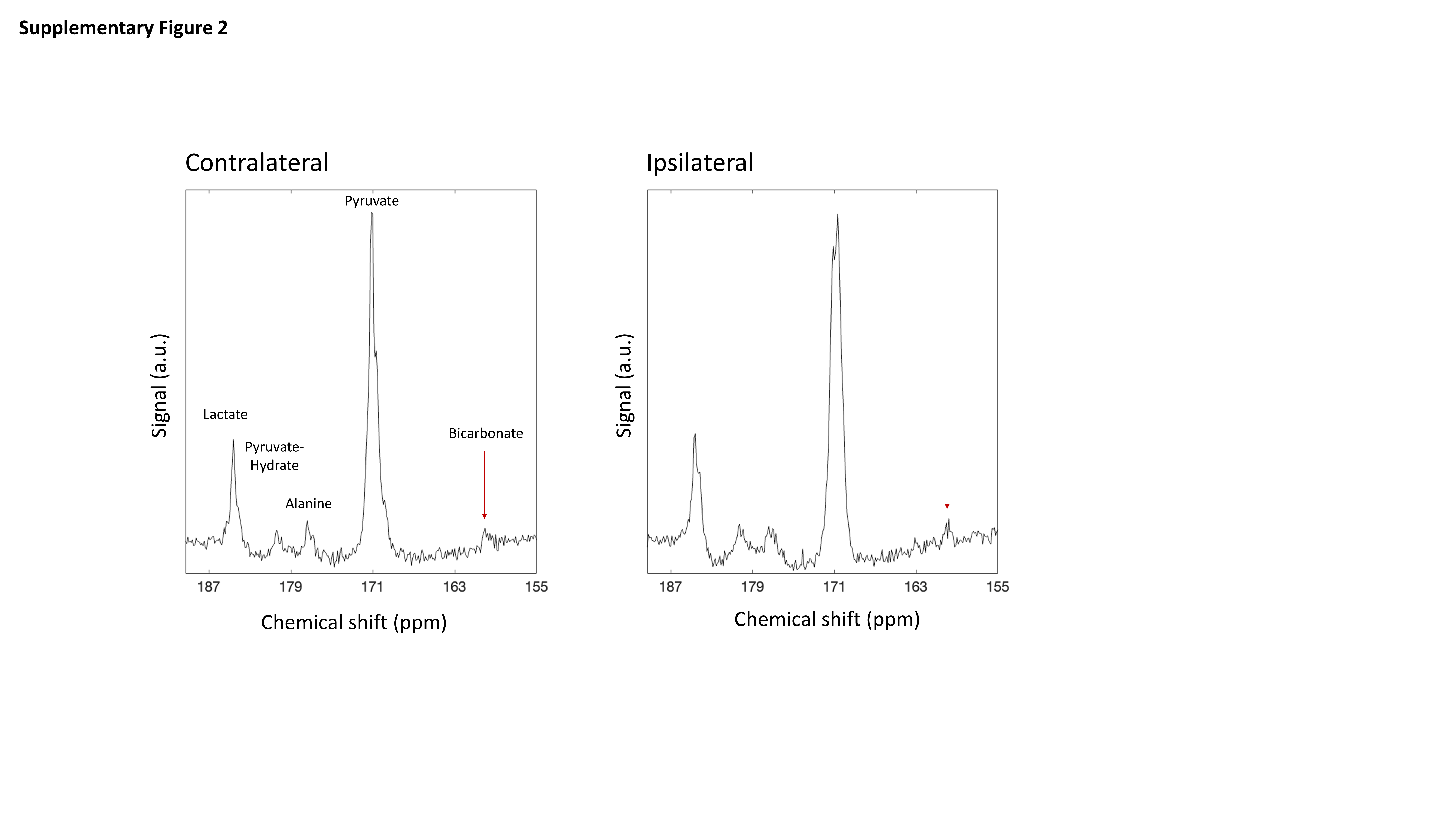

Supplement: Supplementary file 2 — Supplementary Material 2: Fig. S2. Hyperpolarized [1-¹³C]pyruvate spectra from saline-treated control animals. Representative hyperpolarized ¹³C spectra acquired from the ipsilateral and contralateral hemispheres of saline-injected animals. Metabolite resonances are labelled (lactate ~185 ppm, pyruvate-hydrate ~181 ppm, alanine ~178 ppm, pyruvate ~171 ppm, bicarbonate ~161 ppm). In contrast to LPS-treated animals (Figure 2E–F), no visible difference in the bicarbonate peak (indicated by red arrow) is observed between hemispheres in control brains. [file 12974_2026_3839_MOESM2_ESM.png]

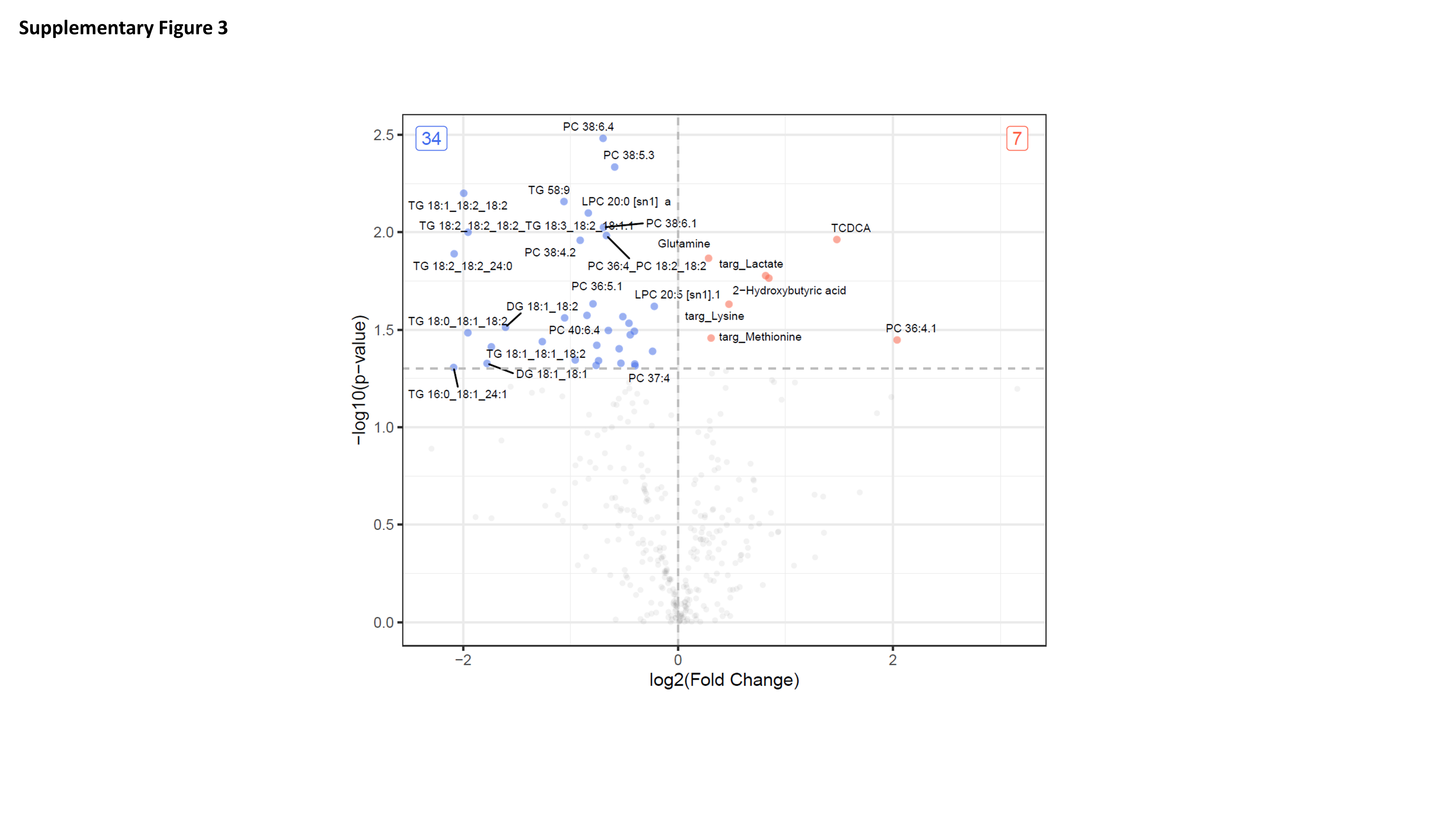

Supplement: Supplementary file 3 — Supplementary Material 3: Fig. S3. Volcano plot of plasma metabolomics data comparing LPS-treated and saline control animals. Volcano plot showing differential plasma metabolites between LPS and saline groups. The x-axis represents log₂ fold-change (LPS vs. saline), and the y-axis represents −log₁₀(p-value). Each point corresponds to a detected metabolite feature (m/z value). Features meeting statistical significance after false discovery rate (FDR) correction are highlighted. Full statistical details, including raw p-values, FDR-adjusted p-values, and fold-changes, are provided in Supplementary Tables 1 and 2. [file 12974_2026_3839_MOESM3_ESM.png]

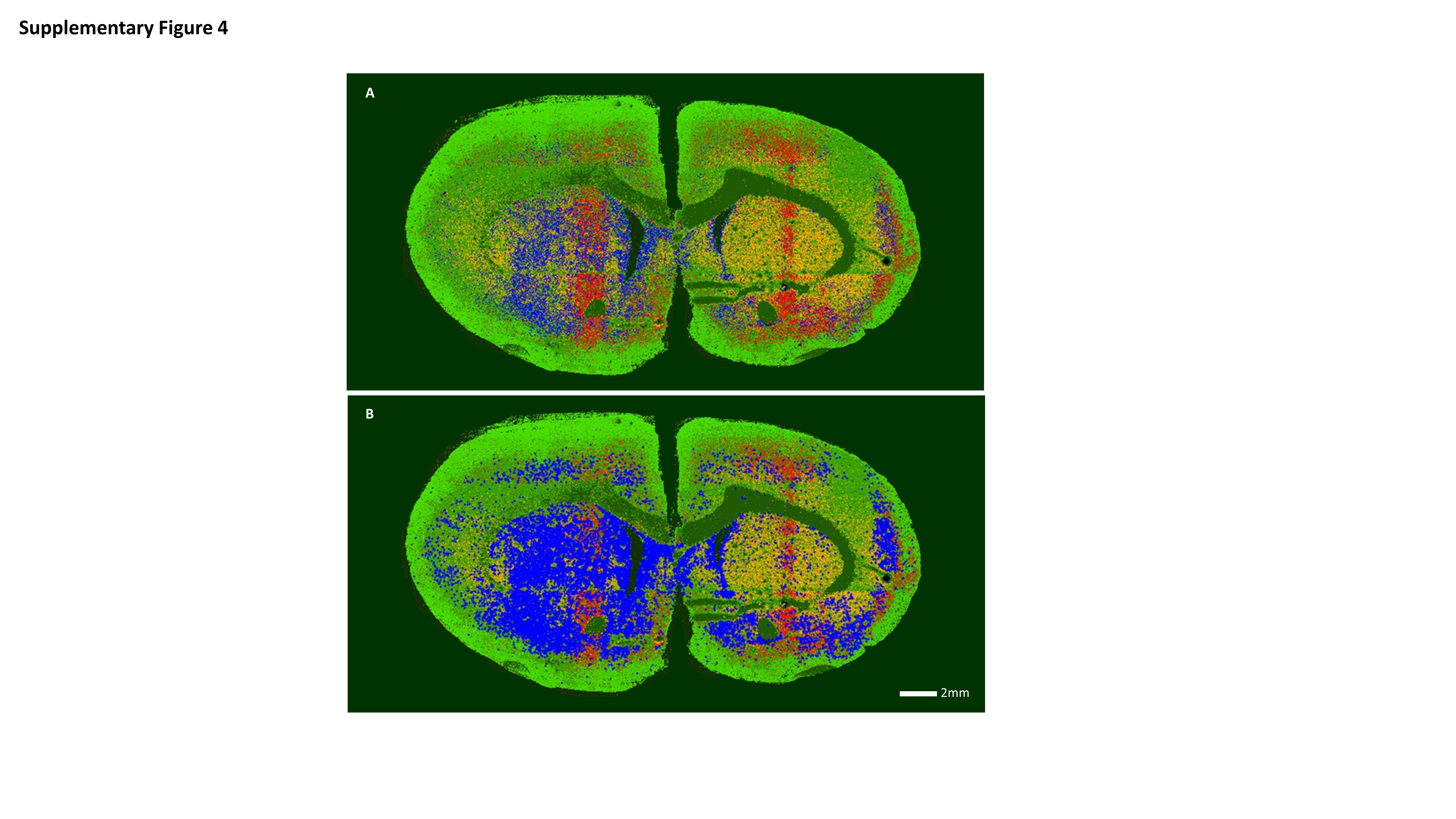

Supplement: Supplementary file 4 — Supplementary Material 4: Fig. S4. Segmentation demonstrates that inflammation induces hemisphere-wise changes in the rat brain. A representative tissue section was selected and subjected to segmentation using the bisecting k-means clustering algorithm, with correlation distance employed as the similarity metric. A - Segmentation map showing the resulting clusters in a representative inflammation-induced tissue section. B - The same segmentation map with the segment of interest highlighted. This segment was further used as an ROI for subsequent co-localization analysis of features relative to the ROI. [file 12974_2026_3839_MOESM4_ESM.png]

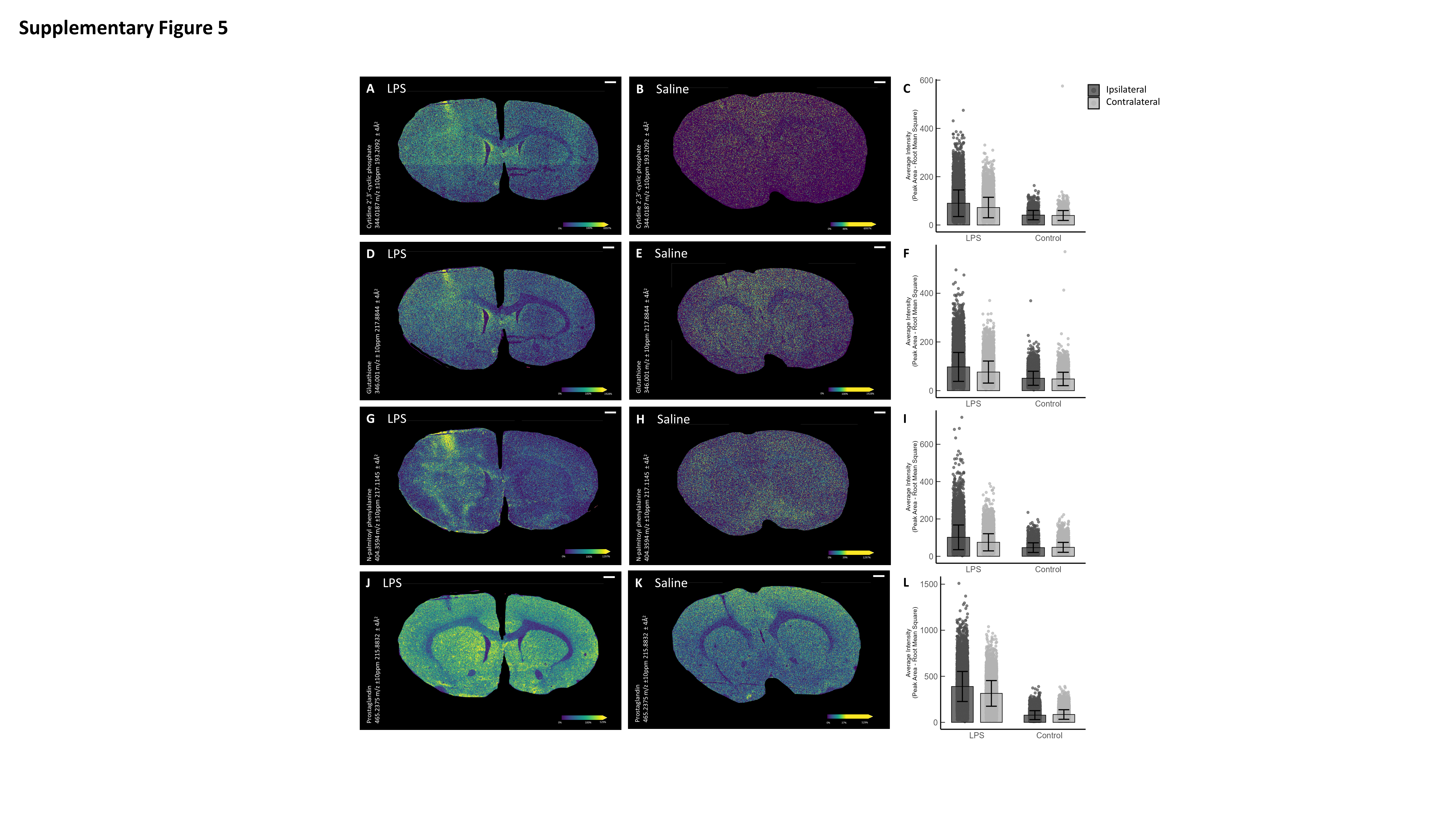

Supplement: Supplementary file 5 — Supplementary Material 5: Fig. S5. LPS-induced neuroinflammation results in ipsilateral changes in selected metabolites. Representative images are high spatial resolution (20µm) root mean square normalized ion images of LPS and saline injected brains for cytidine-2’3-cyclic phosphate (A & B), glutathione (D & E), N-palmitoyl phenylalanine (G & H) and prostaglandin (J & K). The brightness scale is adjusted sample-wise to assess sample to sample intensity variations. Graphs show average distribution of intensities of selected peak after root mean square normalization within the striatum (defined ROI) for cytidine-2’3-cyclic phosphate (C), glutathione (F), N-palmitoyl phenylalanine (I) and prostaglandin (L). Individual data points are shown, with the group mean indicated by a horizontal line. Colour scale represents relative pixel intensity expressed as a percentage of the mean RMS-normalised intensity for that metabolite across the ROI (100% = section mean). Scale bar on representative images shows 800µm. [file 12974_2026_3839_MOESM5_ESM.png]
